# Crystal structure of 1-(2,4-di­methyl­phen­yl)-2-(4-tri­methyl­silyl-1*H*-1,2,3-triazol-1-yl)ethanone

**DOI:** 10.1107/S1600536814024313

**Published:** 2014-11-12

**Authors:** G. B. Venkatesh, H. Nagarajaiah, N. L. Prasad, S. HariPrasad, Noor Shahina Begum

**Affiliations:** a Department of Studies in Chemistry, Bangalore University, Bangalore 560 001, India

**Keywords:** crystal structure, 1,2,3-triazole, tri­methyl­sil­yl, hydrogen bonding, C—H⋯π inter­actions

## Abstract

The asymmetric unit of the title compound, C_15_H_21_N_3_OSi, contains two mol­ecules with similar conformations (r.m.s. overlay fit for the 20 non-H atoms = 0.163 Å). The dihedral angles between the planes of the 1,2,3-triazole and 2,4-di­methyl­benzene rings are 27.0 (3) and 19.5 (3)°. In the crystal, mol­ecules are linked by very weak C—H⋯O and C—H⋯N hydrogen bonds to generate [100] chains. The chains are cross-linked by C—H⋯π inter­actions.

## Related literature   

For related structures and background to the reactions and properties of triazole derivatives, see: Begum *et al.* (2004 ▶); Islor *et al.* (2012 ▶).
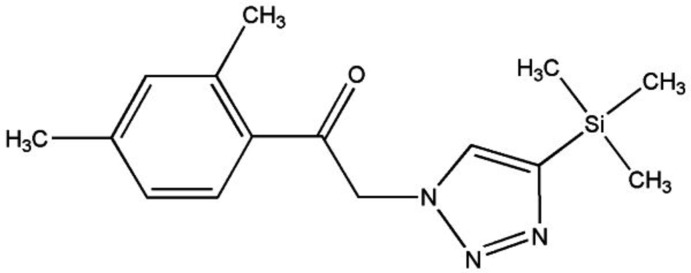


## Experimental   

### Crystal data  

C_15_H_21_N_3_OSi
*M* *_r_* = 287.44
Triclinic, 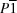

*a* = 5.961 (3) Å
*b* = 13.374 (7) Å
*c* = 20.349 (11) Å
α = 79.034 (10)°
β = 84.831 (10)°
γ = 85.123 (10)°
*V* = 1582.5 (15) Å^3^
*Z* = 4
Mo *K*α radiation
μ = 0.15 mm^−1^
*T* = 100 K
0.18 × 0.15 × 0.12 mm

### Data collection  

Bruker SMART APEX CCD diffractometer
Absorption correction: multi-scan (*SADABS*; Bruker, 1998 ▶) *T*_min_ = 0.974, *T*_max_ = 0.977
9496 measured reflections
6652 independent reflections
2630 reflections with *I* > 2σ(*I*)
*R*_int_ = 0.066

### Refinement  

*R*[*F* ^2^ > 2σ(*F* ^2^)] = 0.092
*wR*(*F* ^2^) = 0.268
*S* = 1.03
6652 reflections
371 parameters
H-atom parameters constrained
Δρ_max_ = 0.59 e Å^−3^
Δρ_min_ = −0.67 e Å^−3^

### 

Data collection: *SMART* (Bruker, 1998 ▶); cell refinement: *SAINT-Plus* (Bruker, 1998 ▶); data reduction: *SAINT-Plus*; program(s) used to solve structure: *SHELXS97* (Sheldrick, 2008 ▶); program(s) used to refine structure: *SHELXL97* (Sheldrick, 2008 ▶); molecular graphics: *ORTEP-3 for Windows* (Farrugia, 2012 ▶) and *CAMERON* (Watkin *et al.*, 1996 ▶); software used to prepare material for publication: *WinGX* (Farrugia, 2012 ▶).

## Supplementary Material

Crystal structure: contains datablock(s) global, I. DOI: 10.1107/S1600536814024313/hb7308sup1.cif

Structure factors: contains datablock(s) I. DOI: 10.1107/S1600536814024313/hb7308Isup2.hkl

Supporting information file. DOI: 10.1107/S1600536814024313/hb7308Isup3.cml

**Fig. 1.**
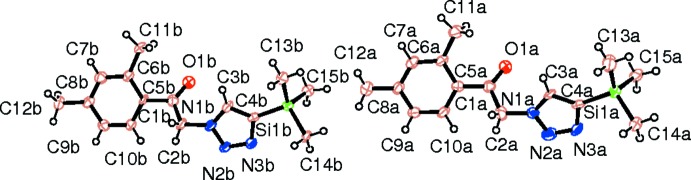
ORTEP view of the title compound, showing 50% probability ellipsoids.

ORTEP . DOI: 10.1107/S1600536814024313/hb7308fig1.tif
*ORTEP* view of the title compound, showing 50% probability ellipsoids.

**Fig. 2.**
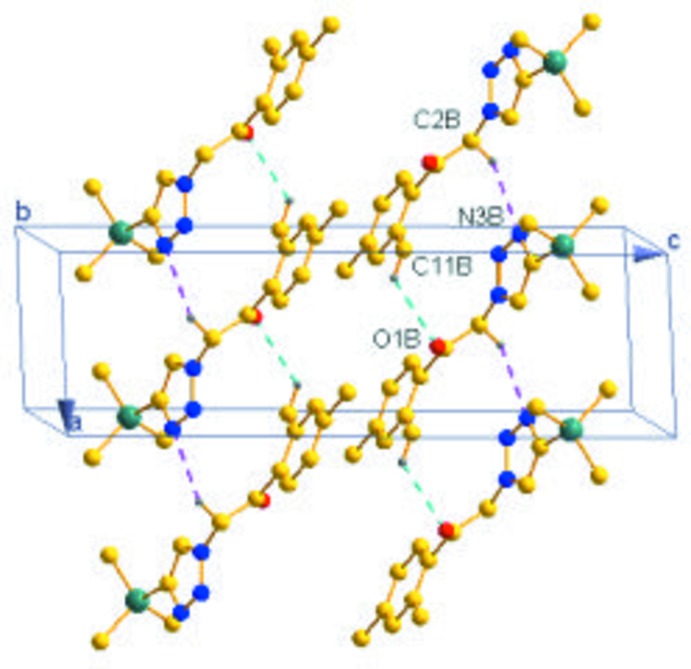
The unit cell packing of the title compound, showing C—H···O and C—H···N interactions. H-atoms not involved in hydrogen bonding have been excluded.

. DOI: 10.1107/S1600536814024313/hb7308fig2.tif
The unit cell packing of the title compound, showing C—H⋯O and C—H⋯N inter­actions. H-atoms not involved in hydrogen bonding have been excluded.

CCDC reference: 1032714

Additional supporting information:  crystallographic information; 3D view; checkCIF report

## Figures and Tables

**Table 1:** Hydrogen-bond geometry (, ) *Cg*1 is the centroid of the C5*B*C10*B* ring.

*D*H*A*	*D*H	H*A*	*D* *A*	*D*H*A*
C11*B*H11*D*O1*B* ^i^	0.98	2.67	3.358(5)	128
C2*B*H2*B*1N3*B* ^i^	0.99	2.66	3.418(7)	134
C12*B*H12*F* *Cg*1^ii^	0.98	2.59	3.446(4)	146

Symmetry codes: (i) 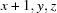; (ii) 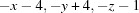.

## supplementary crystallographic information

### S1. Comment

The title compound crystallizes in the triclinic crystal system with space group *P*-1 containing two molecules in the asymmetric unit. The 1,2,3-triazole ring is planar and it is substituted with trimethylsilyl group at one end. The silicon atom has almost tetrahedral coordination with the Si—C distances having characteristic values of 1.848 Å on average. On the other hand, the ring is bridged with 2,4-dimethylbenzene ring through methylenecarbonyl group with a dihedral angle of 27.00 (2)°. The methylene bridged carbonyl group at N1B adopts *cis* configuration with respect to N1═C3B bond because of intramolecular hydrogen bond between carbonyl group of ketone and methyl substituent at C6B along with C3B—H3B of triazole. This locks the molecular conformation and eliminates conformational flexibility. For crystal structure related to the title compound, see: Begum *et al.* (2004) & Islor *et al.* (2012).

The crystal structure of the compound features C—H···O and C—H···N interactions. Both C11B—H11D···O1B and C2B—H2B1···N3B interactions resulted in zigzag one dimensional chain (Figure 2). The crystal structure also features C—H···π interaction of the type C12B—H12F···*Cg* (*Cg* is the centroid of phenyl ring C5B–C10B) 2.59 Å (Table 1).

### S2. Experimental

To a solution of 2-bromo-1-(2,4-dimethylphenyl)ethanone (1 mmol), sodium azide (1.5 mmol) and trimethylsilylacetylene (1.2 mmol) in 30 ml Acetone/H_2_O (1:1 *v*/*v*) was added Cu(OAc)_2_. H_2_O (15 mol%) and sodium ascorbate (30 mol%). The mixture was subjected to ultrasonication for 60 min at room temperature. After completion of the reaction, as indicated by TLC, the reaction mixture was extracted with ethyl acetate. The combined organic layer was washed with brine solution. The organic layer was dried over anhydrous Na_2_SO_4_. The solvent was removed *in vacuo* and the residue was purified by column chromatography to isolate the title compound. Colourless blocks were grown from ethyl acetate solution; Yield: 92%; m.p: 379–381 K; FT–IR (KBr, cm^-1^): 2960 (C—H), 1706 (C═O), 1610 (C═C), 1251 (Si—C); ^1^H NMR (400 MHz, DMSO-d_6_): δ 0.27 (s, 9H), 2.34 (s, 3H), 2.41 (s, 3H), 6.02 (s, 2H), 7.22–7.20 (d, *J* = 8.1 Hz, 2H), 7.95 (s, 1H), 8.07 (s, 1H); ^13^C NMR (400 MHz, CDCl_3_): δ -1.1, 21.4, 21.8, 56.0, 126.7, 129.1, 130.7, 130.8, 133.6, 140.5, 144.0, 146.8, 192.2; ESI–MS *m*/*z*: 288 [M+H]^+^.

### S3. Refinement

The H atoms were placed at calculated positions in the riding model approximation with C—H = 0.95, 0.98, and 0.99 Å for aryl, methyl, and methylene H-atoms respectively, with *U*_iso_(H) = 1.5*U*_eq_(C) for methyl H atoms and *U*_iso_(H) = 1.2*U*_eq_(C) for other H atom.

### Crystal data

**Table:**

C_15_H_21_N_3_OSi	*Z* = 4
*M**_r_* = 287.44	*F*(000) = 616
Triclinic, *P*1	*D*_x_ = 1.206 Mg m^−^^3^
*a* = 5.961 (3) Å	Mo *K*α radiation, λ = 0.71073 Å
*b* = 13.374 (7) Å	θ = 1.0–27.0°
*c* = 20.349 (11) Å	µ = 0.15 mm^−^^1^
α = 79.034 (10)°	*T* = 100 K
β = 84.831 (10)°	Block, colorless
γ = 85.123 (10)°	0.18 × 0.15 × 0.12 mm
*V* = 1582.5 (15) Å^3^	

### Data collection

**Table:**

Bruker SMART APEX CCD diffractometer	6652 independent reflections
Radiation source: fine-focus sealed tube	2630 reflections with *I* > 2σ(*I*)
Graphite monochromator	*R*_int_ = 0.066
ω scans	θ_max_ = 27.0°, θ_min_ = 1.0°
Absorption correction: multi-scan (*SADABS*; Bruker, 1998)	*h* = −7→7
*T*_min_ = 0.974, *T*_max_ = 0.977	*k* = −14→17
9496 measured reflections	*l* = −23→25

### Refinement

**Table:**

Refinement on *F*^2^	Primary atom site location: structure-invariant direct methods
Least-squares matrix: full	Secondary atom site location: difference Fourier map
*R*[*F*^2^ > 2σ(*F*^2^)] = 0.092	Hydrogen site location: inferred from neighbouring sites
*wR*(*F*^2^) = 0.268	H-atom parameters constrained
*S* = 1.03	*w* = 1/[σ^2^(*F*_o_^2^) + (0.0941*P*)^2^] where *P* = (*F*_o_^2^ + 2*F*_c_^2^)/3
6652 reflections	(Δ/σ)_max_ = 0.004
371 parameters	Δρ_max_ = 0.59 e Å^−^^3^
0 restraints	Δρ_min_ = −0.67 e Å^−^^3^

### Special details

**Table:**

Geometry. All s.u.'s (except the s.u. in the dihedral angle between two l.s. planes) are estimated using the full covariance matrix. The cell s.u.'s are taken into account individually in the estimation of s.u.'s in distances, angles and torsion angles; correlations between s.u.'s in cell parameters are only used when they are defined by crystal symmetry. An approximate (isotropic) treatment of cell s.u.'s is used for estimating s.u.'s involving l.s. planes.
Refinement. Refinement of *F*^2^ against ALL reflections. The weighted *R*-factor *wR* and goodness of fit *S* are based on *F*^2^, conventional *R*-factors *R* are based on *F*, with *F* set to zero for negative *F*^2^. The threshold expression of *F*^2^ > 2σ(*F*^2^) is used only for calculating *R*-factors(gt) *etc*. and is not relevant to the choice of reflections for refinement. *R*-factors based on *F*^2^ are statistically about twice as large as those based on *F*, and *R*- factors based on ALL data will be even larger.

### Fractional atomic coordinates and isotropic or equivalent isotropic displacement parameters (Å^2^)

**Table:**

	*x*	*y*	*z*	*U*_iso_*/*U*_eq_	
Si1A	0.6655 (3)	0.63732 (13)	0.37769 (8)	0.0357 (5)	
O1A	1.1714 (7)	0.8439 (3)	0.1518 (2)	0.0434 (11)	
N1A	0.8973 (9)	0.8892 (4)	0.2595 (2)	0.0390 (13)	
N2A	0.6708 (9)	0.9139 (4)	0.2642 (2)	0.0437 (14)	
N3A	0.5761 (8)	0.8349 (4)	0.3016 (2)	0.0379 (13)	
C1A	1.1885 (9)	0.9286 (5)	0.1633 (3)	0.0321 (14)	
C2A	1.0499 (11)	0.9633 (5)	0.2231 (3)	0.0445 (17)	
H2A1	1.1548	0.9781	0.2545	0.053*	
H2A2	0.9606	1.0277	0.2067	0.053*	
C3A	0.9454 (9)	0.7958 (4)	0.2940 (3)	0.0327 (14)	
H3A	1.0910	0.7614	0.2983	0.039*	
C4A	0.7423 (9)	0.7585 (4)	0.3223 (3)	0.0308 (14)	
C5A	1.3451 (10)	1.0026 (4)	0.1252 (3)	0.0304 (14)	
C6A	1.5553 (9)	0.9695 (5)	0.0966 (3)	0.0315 (14)	
C7A	1.6955 (10)	1.0443 (5)	0.0638 (3)	0.0383 (15)	
H7A	1.8401	1.0229	0.0456	0.046*	
C8A	1.6372 (10)	1.1479 (5)	0.0560 (3)	0.0353 (15)	
C9A	1.4301 (10)	1.1781 (5)	0.0831 (3)	0.0361 (15)	
H9A	1.3848	1.2488	0.0782	0.043*	
C10A	1.2854 (9)	1.1069 (5)	0.1177 (3)	0.0342 (15)	
H10A	1.1431	1.1297	0.1366	0.041*	
C11A	1.6318 (10)	0.8584 (4)	0.1027 (3)	0.0433 (16)	
H11A	1.7949	0.8514	0.0913	0.065*	
H11B	1.5529	0.8280	0.0719	0.065*	
H11C	1.5976	0.8231	0.1489	0.065*	
C12A	1.7946 (10)	1.2257 (5)	0.0189 (3)	0.0488 (18)	
H12A	1.9511	1.1972	0.0220	0.073*	
H12B	1.7738	1.2873	0.0388	0.073*	
H12C	1.7613	1.2431	−0.0284	0.073*	
C13A	0.9173 (10)	0.5666 (5)	0.4104 (3)	0.0508 (18)	
H13A	1.0211	0.5506	0.3731	0.076*	
H13B	0.8756	0.5031	0.4400	0.076*	
H13C	0.9914	0.6080	0.4358	0.076*	
C14A	0.4733 (10)	0.6683 (5)	0.4500 (3)	0.0448 (17)	
H14A	0.5455	0.7141	0.4726	0.067*	
H14B	0.4426	0.6052	0.4817	0.067*	
H14C	0.3312	0.7017	0.4335	0.067*	
C15A	0.5243 (10)	0.5607 (5)	0.3288 (3)	0.0451 (17)	
H15A	0.3985	0.6023	0.3072	0.068*	
H15B	0.4668	0.5007	0.3589	0.068*	
H15C	0.6329	0.5387	0.2944	0.068*	
Si1B	2.0301 (3)	1.48939 (13)	−0.13801 (8)	0.0339 (5)	
O1B	2.6087 (7)	1.6995 (3)	−0.3364 (2)	0.0432 (11)	
N1B	2.3335 (8)	1.7427 (4)	−0.2292 (2)	0.0333 (12)	
N2B	2.1155 (8)	1.7787 (4)	−0.2235 (2)	0.0363 (12)	
N3B	1.9970 (8)	1.7011 (4)	−0.1939 (2)	0.0361 (12)	
C1B	2.6300 (10)	1.7819 (5)	−0.3206 (3)	0.0325 (14)	
C2B	2.5058 (9)	1.8107 (4)	−0.2585 (3)	0.0341 (15)	
H2B1	2.6156	1.8109	−0.2248	0.041*	
H2B2	2.4351	1.8809	−0.2700	0.041*	
C3B	2.3521 (9)	1.6422 (4)	−0.2044 (3)	0.0311 (14)	
H3B	2.4876	1.5993	−0.2033	0.037*	
C4B	2.1414 (9)	1.6127 (4)	−0.1812 (3)	0.0296 (13)	
C5B	2.7930 (8)	1.8559 (4)	−0.3571 (3)	0.0277 (13)	
C6B	3.0050 (9)	1.8236 (4)	−0.3865 (3)	0.0320 (14)	
C7B	3.1501 (10)	1.8981 (5)	−0.4176 (3)	0.0361 (15)	
H7B	3.2941	1.8772	−0.4364	0.043*	
C8B	3.0914 (10)	2.0014 (5)	−0.4219 (3)	0.0332 (14)	
C9B	2.8835 (10)	2.0317 (5)	−0.3945 (3)	0.0353 (15)	
H9B	2.8398	2.1024	−0.3981	0.042*	
C10B	2.7368 (9)	1.9601 (4)	−0.3617 (3)	0.0322 (14)	
H10B	2.5956	1.9825	−0.3419	0.039*	
C11B	3.0815 (10)	1.7114 (4)	−0.3847 (3)	0.0395 (16)	
H11D	3.2394	1.7058	−0.4026	0.059*	
H11E	2.9869	1.6822	−0.4122	0.059*	
H11F	3.0675	1.6742	−0.3383	0.059*	
C12B	3.2525 (9)	2.0802 (5)	−0.4572 (3)	0.0377 (15)	
H12D	3.4009	2.0641	−0.4392	0.056*	
H12E	3.1935	2.1482	−0.4499	0.056*	
H12F	3.2671	2.0793	−0.5055	0.056*	
C13B	2.2658 (10)	1.4065 (5)	−0.1010 (3)	0.0415 (16)	
H13D	2.3782	1.3920	−0.1367	0.062*	
H13E	2.2093	1.3424	−0.0759	0.062*	
H13F	2.3357	1.4410	−0.0704	0.062*	
C14B	1.8129 (8)	1.5216 (4)	−0.0720 (3)	0.0342 (15)	
H14D	1.8788	1.5617	−0.0440	0.051*	
H14E	1.7613	1.4585	−0.0440	0.051*	
H14F	1.6846	1.5614	−0.0933	0.051*	
C15B	1.8988 (10)	1.4271 (5)	−0.1982 (3)	0.0454 (17)	
H15D	1.8027	1.4780	−0.2262	0.068*	
H15E	1.8073	1.3728	−0.1732	0.068*	
H15F	2.0176	1.3978	−0.2269	0.068*	

### Atomic displacement parameters (Å^2^)

**Table:**

	*U*^11^	*U*^22^	*U*^33^	*U*^12^	*U*^13^	*U*^23^
Si1A	0.0272 (9)	0.0354 (11)	0.0438 (10)	−0.0046 (8)	0.0054 (8)	−0.0080 (8)
O1A	0.040 (3)	0.034 (3)	0.058 (3)	−0.008 (2)	0.009 (2)	−0.016 (2)
N1A	0.044 (3)	0.028 (3)	0.041 (3)	0.001 (2)	0.016 (3)	−0.006 (2)
N2A	0.040 (3)	0.043 (4)	0.042 (3)	0.010 (3)	0.002 (3)	−0.003 (3)
N3A	0.026 (3)	0.044 (3)	0.039 (3)	−0.002 (2)	0.011 (2)	−0.004 (3)
C1A	0.026 (3)	0.033 (4)	0.036 (3)	0.001 (3)	−0.004 (3)	−0.004 (3)
C2A	0.054 (4)	0.033 (4)	0.043 (4)	−0.012 (3)	0.014 (3)	−0.005 (3)
C3A	0.023 (3)	0.038 (4)	0.034 (3)	0.002 (3)	0.016 (3)	−0.007 (3)
C4A	0.024 (3)	0.029 (3)	0.038 (3)	0.009 (3)	0.004 (3)	−0.010 (3)
C5A	0.030 (3)	0.028 (4)	0.032 (3)	0.002 (3)	−0.003 (3)	−0.006 (3)
C6A	0.021 (3)	0.037 (4)	0.035 (3)	0.004 (3)	0.002 (3)	−0.008 (3)
C7A	0.021 (3)	0.045 (4)	0.048 (4)	0.001 (3)	−0.002 (3)	−0.008 (3)
C8A	0.030 (3)	0.042 (4)	0.033 (3)	−0.005 (3)	0.007 (3)	−0.007 (3)
C9A	0.035 (4)	0.029 (4)	0.041 (4)	0.004 (3)	0.006 (3)	−0.005 (3)
C10A	0.023 (3)	0.042 (4)	0.034 (3)	0.003 (3)	0.008 (3)	−0.006 (3)
C11A	0.033 (4)	0.043 (4)	0.055 (4)	0.004 (3)	−0.003 (3)	−0.013 (3)
C12A	0.035 (4)	0.047 (4)	0.065 (4)	−0.007 (3)	0.004 (4)	−0.010 (4)
C13A	0.039 (4)	0.050 (5)	0.058 (4)	−0.008 (3)	−0.003 (3)	0.006 (3)
C14A	0.034 (4)	0.044 (4)	0.056 (4)	−0.009 (3)	0.013 (3)	−0.014 (3)
C15A	0.022 (3)	0.050 (4)	0.064 (4)	−0.003 (3)	0.005 (3)	−0.018 (4)
Si1B	0.0269 (9)	0.0306 (10)	0.0421 (10)	0.0004 (7)	0.0058 (8)	−0.0065 (8)
O1B	0.037 (3)	0.038 (3)	0.055 (3)	−0.004 (2)	0.005 (2)	−0.013 (2)
N1B	0.020 (3)	0.037 (3)	0.040 (3)	0.002 (2)	0.004 (2)	−0.004 (2)
N2B	0.025 (3)	0.029 (3)	0.050 (3)	0.003 (2)	0.012 (2)	−0.003 (2)
N3B	0.028 (3)	0.029 (3)	0.046 (3)	0.000 (2)	0.008 (2)	−0.001 (2)
C1B	0.028 (3)	0.030 (4)	0.038 (3)	0.000 (3)	−0.007 (3)	0.002 (3)
C2B	0.018 (3)	0.038 (4)	0.042 (3)	−0.004 (3)	0.008 (3)	−0.002 (3)
C3B	0.018 (3)	0.039 (4)	0.031 (3)	0.001 (3)	0.004 (3)	0.002 (3)
C4B	0.019 (3)	0.031 (3)	0.038 (3)	0.006 (2)	0.000 (3)	−0.007 (3)
C5B	0.011 (3)	0.035 (4)	0.035 (3)	−0.002 (2)	0.004 (2)	−0.003 (3)
C6B	0.022 (3)	0.036 (4)	0.038 (3)	−0.001 (3)	−0.001 (3)	−0.007 (3)
C7B	0.034 (4)	0.036 (4)	0.037 (3)	0.000 (3)	−0.004 (3)	−0.004 (3)
C8B	0.027 (3)	0.041 (4)	0.029 (3)	−0.002 (3)	0.007 (3)	−0.005 (3)
C9B	0.037 (4)	0.030 (4)	0.038 (3)	−0.004 (3)	0.002 (3)	−0.005 (3)
C10B	0.022 (3)	0.031 (4)	0.042 (3)	−0.001 (3)	−0.001 (3)	−0.005 (3)
C11B	0.024 (3)	0.044 (4)	0.049 (4)	0.002 (3)	0.009 (3)	−0.012 (3)
C12B	0.027 (3)	0.043 (4)	0.042 (4)	−0.010 (3)	0.001 (3)	−0.004 (3)
C13B	0.038 (4)	0.036 (4)	0.048 (4)	−0.004 (3)	0.010 (3)	−0.006 (3)
C14B	0.012 (3)	0.037 (4)	0.050 (4)	−0.001 (2)	0.004 (3)	−0.003 (3)
C15B	0.033 (4)	0.041 (4)	0.064 (4)	−0.004 (3)	0.009 (3)	−0.019 (3)

### Geometric parameters (Å, º)

**Table:**

Si1A—C13A	1.814 (6)	Si1B—C13B	1.839 (6)
Si1A—C15A	1.853 (6)	Si1B—C4B	1.860 (6)
Si1A—C4A	1.856 (6)	Si1B—C14B	1.864 (6)
Si1A—C14A	1.870 (6)	Si1B—C15B	1.865 (6)
O1A—C1A	1.214 (7)	O1B—C1B	1.224 (7)
N1A—C3A	1.333 (7)	N1B—C3B	1.341 (7)
N1A—N2A	1.362 (7)	N1B—N2B	1.350 (6)
N1A—C2A	1.453 (7)	N1B—C2B	1.442 (6)
N2A—N3A	1.317 (6)	N2B—N3B	1.322 (6)
N3A—C4A	1.394 (7)	N3B—C4B	1.399 (7)
C1A—C5A	1.478 (8)	C1B—C5B	1.493 (7)
C1A—C2A	1.532 (8)	C1B—C2B	1.504 (8)
C2A—H2A1	0.9900	C2B—H2B1	0.9900
C2A—H2A2	0.9900	C2B—H2B2	0.9900
C3A—C4A	1.383 (7)	C3B—C4B	1.365 (7)
C3A—H3A	0.9500	C3B—H3B	0.9500
C5A—C10A	1.394 (8)	C5B—C10B	1.393 (8)
C5A—C6A	1.406 (8)	C5B—C6B	1.418 (7)
C6A—C7A	1.390 (7)	C6B—C7B	1.397 (7)
C6A—C11A	1.503 (8)	C6B—C11B	1.526 (8)
C7A—C8A	1.383 (8)	C7B—C8B	1.384 (8)
C7A—H7A	0.9500	C7B—H7B	0.9500
C8A—C9A	1.367 (8)	C8B—C9B	1.374 (8)
C8A—C12A	1.507 (8)	C8B—C12B	1.520 (7)
C9A—C10A	1.385 (7)	C9B—C10B	1.387 (7)
C9A—H9A	0.9500	C9B—H9B	0.9500
C10A—H10A	0.9500	C10B—H10B	0.9500
C11A—H11A	0.9800	C11B—H11D	0.9800
C11A—H11B	0.9800	C11B—H11E	0.9800
C11A—H11C	0.9800	C11B—H11F	0.9800
C12A—H12A	0.9800	C12B—H12D	0.9800
C12A—H12B	0.9800	C12B—H12E	0.9800
C12A—H12C	0.9800	C12B—H12F	0.9800
C13A—H13A	0.9800	C13B—H13D	0.9800
C13A—H13B	0.9800	C13B—H13E	0.9800
C13A—H13C	0.9800	C13B—H13F	0.9800
C14A—H14A	0.9800	C14B—H14D	0.9800
C14A—H14B	0.9800	C14B—H14E	0.9800
C14A—H14C	0.9800	C14B—H14F	0.9800
C15A—H15A	0.9800	C15B—H15D	0.9800
C15A—H15B	0.9800	C15B—H15E	0.9800
C15A—H15C	0.9800	C15B—H15F	0.9800
			
C13A—Si1A—C15A	110.1 (3)	C13B—Si1B—C4B	108.2 (3)
C13A—Si1A—C4A	109.8 (3)	C13B—Si1B—C14B	111.4 (3)
C15A—Si1A—C4A	109.0 (3)	C4B—Si1B—C14B	106.0 (3)
C13A—Si1A—C14A	108.4 (3)	C13B—Si1B—C15B	110.4 (3)
C15A—Si1A—C14A	110.7 (3)	C4B—Si1B—C15B	110.8 (3)
C4A—Si1A—C14A	108.7 (3)	C14B—Si1B—C15B	110.1 (3)
C3A—N1A—N2A	110.8 (5)	C3B—N1B—N2B	110.0 (4)
C3A—N1A—C2A	129.2 (5)	C3B—N1B—C2B	129.8 (5)
N2A—N1A—C2A	120.0 (5)	N2B—N1B—C2B	120.2 (5)
N3A—N2A—N1A	106.9 (5)	N3B—N2B—N1B	107.2 (5)
N2A—N3A—C4A	109.6 (5)	N2B—N3B—C4B	109.4 (5)
O1A—C1A—C5A	124.6 (6)	O1B—C1B—C5B	123.2 (6)
O1A—C1A—C2A	119.9 (5)	O1B—C1B—C2B	121.2 (5)
C5A—C1A—C2A	115.3 (5)	C5B—C1B—C2B	115.4 (5)
N1A—C2A—C1A	114.0 (5)	N1B—C2B—C1B	113.2 (5)
N1A—C2A—H2A1	108.7	N1B—C2B—H2B1	108.9
C1A—C2A—H2A1	108.7	C1B—C2B—H2B1	108.9
N1A—C2A—H2A2	108.7	N1B—C2B—H2B2	108.9
C1A—C2A—H2A2	108.7	C1B—C2B—H2B2	108.9
H2A1—C2A—H2A2	107.6	H2B1—C2B—H2B2	107.8
N1A—C3A—C4A	106.8 (5)	N1B—C3B—C4B	107.8 (5)
N1A—C3A—H3A	126.6	N1B—C3B—H3B	126.1
C4A—C3A—H3A	126.6	C4B—C3B—H3B	126.1
C3A—C4A—N3A	106.0 (5)	C3B—C4B—N3B	105.6 (5)
C3A—C4A—Si1A	133.5 (5)	C3B—C4B—Si1B	133.8 (5)
N3A—C4A—Si1A	120.5 (4)	N3B—C4B—Si1B	120.6 (4)
C10A—C5A—C6A	119.1 (5)	C10B—C5B—C6B	118.8 (5)
C10A—C5A—C1A	119.8 (5)	C10B—C5B—C1B	119.1 (5)
C6A—C5A—C1A	121.1 (5)	C6B—C5B—C1B	122.2 (5)
C7A—C6A—C5A	117.3 (5)	C7B—C6B—C5B	118.2 (6)
C7A—C6A—C11A	120.5 (5)	C7B—C6B—C11B	118.9 (5)
C5A—C6A—C11A	122.2 (5)	C5B—C6B—C11B	122.8 (5)
C8A—C7A—C6A	124.0 (6)	C8B—C7B—C6B	122.3 (6)
C8A—C7A—H7A	118.0	C8B—C7B—H7B	118.9
C6A—C7A—H7A	118.0	C6B—C7B—H7B	118.9
C9A—C8A—C7A	117.6 (5)	C9B—C8B—C7B	118.9 (5)
C9A—C8A—C12A	120.7 (6)	C9B—C8B—C12B	120.4 (6)
C7A—C8A—C12A	121.7 (6)	C7B—C8B—C12B	120.7 (5)
C8A—C9A—C10A	120.9 (6)	C8B—C9B—C10B	120.6 (6)
C8A—C9A—H9A	119.6	C8B—C9B—H9B	119.7
C10A—C9A—H9A	119.6	C10B—C9B—H9B	119.7
C9A—C10A—C5A	121.2 (6)	C9B—C10B—C5B	121.2 (6)
C9A—C10A—H10A	119.4	C9B—C10B—H10B	119.4
C5A—C10A—H10A	119.4	C5B—C10B—H10B	119.4
C6A—C11A—H11A	109.5	C6B—C11B—H11D	109.5
C6A—C11A—H11B	109.5	C6B—C11B—H11E	109.5
H11A—C11A—H11B	109.5	H11D—C11B—H11E	109.5
C6A—C11A—H11C	109.5	C6B—C11B—H11F	109.5
H11A—C11A—H11C	109.5	H11D—C11B—H11F	109.5
H11B—C11A—H11C	109.5	H11E—C11B—H11F	109.5
C8A—C12A—H12A	109.5	C8B—C12B—H12D	109.5
C8A—C12A—H12B	109.5	C8B—C12B—H12E	109.5
H12A—C12A—H12B	109.5	H12D—C12B—H12E	109.5
C8A—C12A—H12C	109.5	C8B—C12B—H12F	109.5
H12A—C12A—H12C	109.5	H12D—C12B—H12F	109.5
H12B—C12A—H12C	109.5	H12E—C12B—H12F	109.5
Si1A—C13A—H13A	109.5	Si1B—C13B—H13D	109.5
Si1A—C13A—H13B	109.5	Si1B—C13B—H13E	109.5
H13A—C13A—H13B	109.5	H13D—C13B—H13E	109.5
Si1A—C13A—H13C	109.5	Si1B—C13B—H13F	109.5
H13A—C13A—H13C	109.5	H13D—C13B—H13F	109.5
H13B—C13A—H13C	109.5	H13E—C13B—H13F	109.5
Si1A—C14A—H14A	109.5	Si1B—C14B—H14D	109.5
Si1A—C14A—H14B	109.5	Si1B—C14B—H14E	109.5
H14A—C14A—H14B	109.5	H14D—C14B—H14E	109.5
Si1A—C14A—H14C	109.5	Si1B—C14B—H14F	109.5
H14A—C14A—H14C	109.5	H14D—C14B—H14F	109.5
H14B—C14A—H14C	109.5	H14E—C14B—H14F	109.5
Si1A—C15A—H15A	109.5	Si1B—C15B—H15D	109.5
Si1A—C15A—H15B	109.5	Si1B—C15B—H15E	109.5
H15A—C15A—H15B	109.5	H15D—C15B—H15E	109.5
Si1A—C15A—H15C	109.5	Si1B—C15B—H15F	109.5
H15A—C15A—H15C	109.5	H15D—C15B—H15F	109.5
H15B—C15A—H15C	109.5	H15E—C15B—H15F	109.5
			
C3A—N1A—N2A—N3A	−0.4 (6)	C3B—N1B—N2B—N3B	1.1 (6)
C2A—N1A—N2A—N3A	−177.6 (5)	C2B—N1B—N2B—N3B	−178.6 (5)
N1A—N2A—N3A—C4A	0.8 (6)	N1B—N2B—N3B—C4B	−0.8 (6)
C3A—N1A—C2A—C1A	63.9 (8)	C3B—N1B—C2B—C1B	61.0 (8)
N2A—N1A—C2A—C1A	−119.6 (6)	N2B—N1B—C2B—C1B	−119.3 (6)
O1A—C1A—C2A—N1A	−2.9 (8)	O1B—C1B—C2B—N1B	−11.4 (8)
C5A—C1A—C2A—N1A	179.9 (5)	C5B—C1B—C2B—N1B	173.7 (5)
N2A—N1A—C3A—C4A	−0.1 (7)	N2B—N1B—C3B—C4B	−0.9 (6)
C2A—N1A—C3A—C4A	176.7 (5)	C2B—N1B—C3B—C4B	178.8 (5)
N1A—C3A—C4A—N3A	0.6 (6)	N1B—C3B—C4B—N3B	0.4 (6)
N1A—C3A—C4A—Si1A	−178.8 (4)	N1B—C3B—C4B—Si1B	−177.6 (4)
N2A—N3A—C4A—C3A	−0.9 (6)	N2B—N3B—C4B—C3B	0.3 (6)
N2A—N3A—C4A—Si1A	178.6 (4)	N2B—N3B—C4B—Si1B	178.6 (4)
C13A—Si1A—C4A—C3A	11.9 (7)	C13B—Si1B—C4B—C3B	19.6 (7)
C15A—Si1A—C4A—C3A	−108.9 (6)	C14B—Si1B—C4B—C3B	139.1 (6)
C14A—Si1A—C4A—C3A	130.3 (6)	C15B—Si1B—C4B—C3B	−101.5 (6)
C13A—Si1A—C4A—N3A	−167.4 (5)	C13B—Si1B—C4B—N3B	−158.2 (4)
C15A—Si1A—C4A—N3A	71.8 (5)	C14B—Si1B—C4B—N3B	−38.6 (5)
C14A—Si1A—C4A—N3A	−49.0 (5)	C15B—Si1B—C4B—N3B	80.7 (5)
O1A—C1A—C5A—C10A	148.5 (6)	O1B—C1B—C5B—C10B	147.1 (6)
C2A—C1A—C5A—C10A	−34.4 (7)	C2B—C1B—C5B—C10B	−38.1 (7)
O1A—C1A—C5A—C6A	−32.1 (9)	O1B—C1B—C5B—C6B	−33.8 (9)
C2A—C1A—C5A—C6A	144.9 (6)	C2B—C1B—C5B—C6B	140.9 (6)
C10A—C5A—C6A—C7A	1.8 (8)	C10B—C5B—C6B—C7B	1.3 (8)
C1A—C5A—C6A—C7A	−177.6 (5)	C1B—C5B—C6B—C7B	−177.8 (5)
C10A—C5A—C6A—C11A	179.8 (5)	C10B—C5B—C6B—C11B	−179.5 (5)
C1A—C5A—C6A—C11A	0.4 (9)	C1B—C5B—C6B—C11B	1.5 (9)
C5A—C6A—C7A—C8A	−2.1 (9)	C5B—C6B—C7B—C8B	−1.6 (8)
C11A—C6A—C7A—C8A	179.9 (6)	C11B—C6B—C7B—C8B	179.1 (5)
C6A—C7A—C8A—C9A	0.9 (9)	C6B—C7B—C8B—C9B	0.3 (9)
C6A—C7A—C8A—C12A	−178.9 (5)	C6B—C7B—C8B—C12B	−178.9 (5)
C7A—C8A—C9A—C10A	0.6 (9)	C7B—C8B—C9B—C10B	1.5 (9)
C12A—C8A—C9A—C10A	−179.6 (5)	C12B—C8B—C9B—C10B	−179.3 (5)
C8A—C9A—C10A—C5A	−0.8 (9)	C8B—C9B—C10B—C5B	−1.8 (9)
C6A—C5A—C10A—C9A	−0.4 (8)	C6B—C5B—C10B—C9B	0.4 (8)
C1A—C5A—C10A—C9A	179.0 (5)	C1B—C5B—C10B—C9B	179.4 (5)

### Hydrogen-bond geometry (Å, º)

Cg1 is the centroid of the C5B–C10B ring.

**Table:**

*D*—H···*A*	*D*—H	H···*A*	*D*···*A*	*D*—H···*A*
C11*B*—H11*D*···O1*B*^i^	0.98	2.67	3.358 (5)	128
C2*B*—H2*B*1···N3*B*^i^	0.99	2.66	3.418 (7)	134
C12*B*—H12*F*···*Cg*1^ii^	0.98	2.59	3.446 (4)	146

Symmetry codes: (i) *x*+1, *y*, *z*; (ii) −*x*−4, −*y*+4, −*z*−1.
