# Supplementary figures and images for: Crystal structure of 1-(2,4-di­methyl­phen­yl)-2-(4-tri­methyl­silyl-1H-1,2,3-triazol-1-yl)ethanone
Source: Acta Crystallogr Sect E Struct Rep Online. 2014 Nov 12;70(Pt 12):o1253. doi: 10.1107/S1600536814024313 (PMC4257412; doi:10.1107/S1600536814024313)

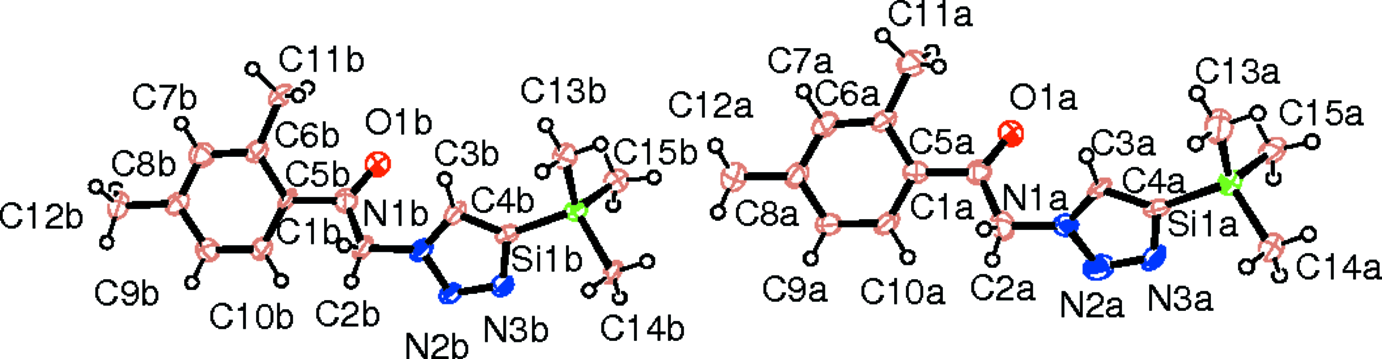

Supplement: Supplementary file 4 [file e-70-o1253-fig1.tif]

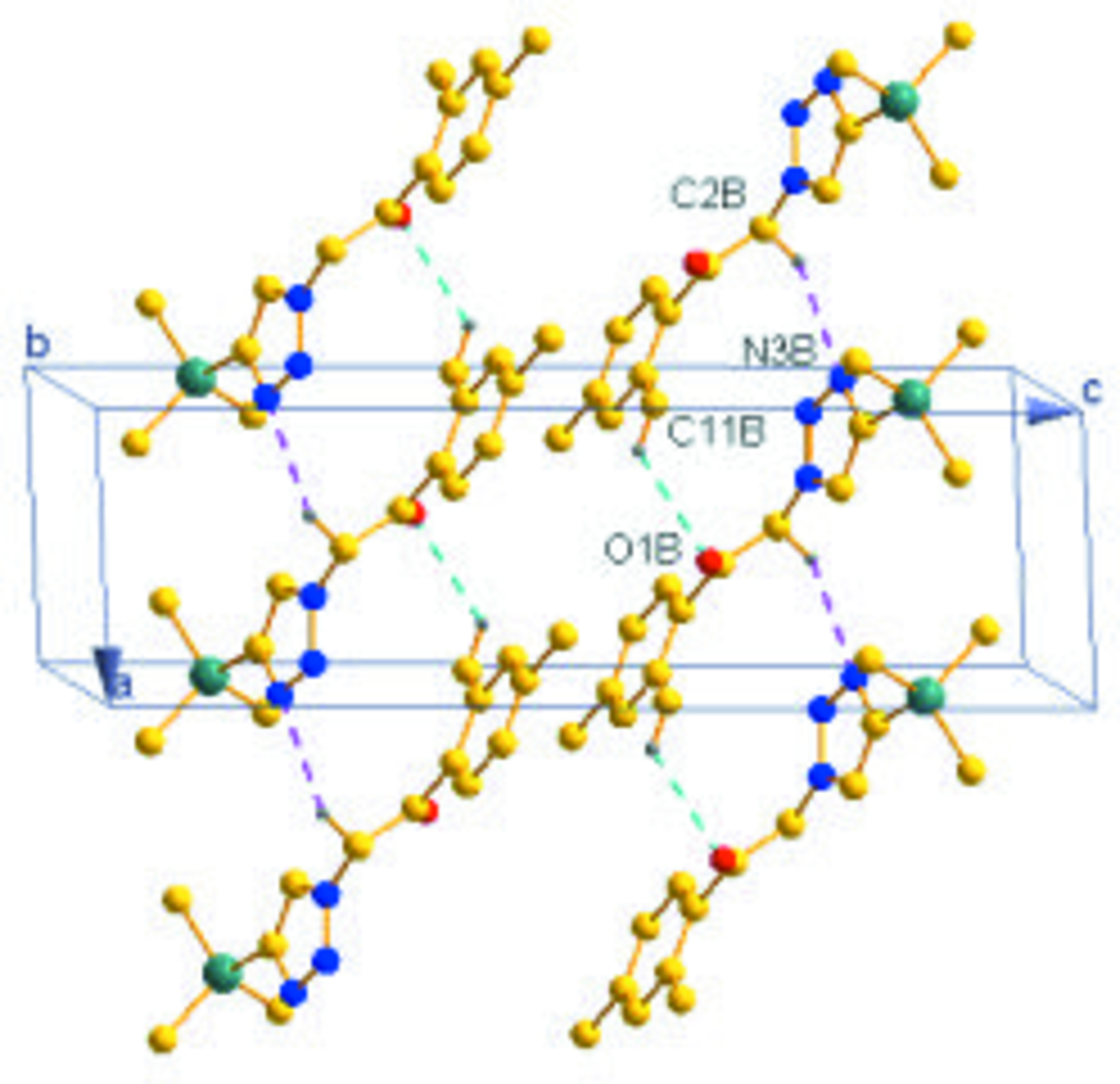

Supplement: Supplementary file 5 [file e-70-o1253-fig2.tif]
